# Effects of Body-Color Mutations on Vitality: An Attempt to Establish Easy-to-Breed See-Through Medaka Strains by Outcrossing

**DOI:** 10.1534/g3.113.007575

**Published:** 2013-09-01

**Authors:** Ayaka Ohshima, Noriko Morimura, Chizuru Matsumoto, Ami Hiraga, Ritsuko Komine, Tetsuaki Kimura, Kiyoshi Naruse, Shoji Fukamachi

**Affiliations:** * Laboratory of Evolutionary Genetics, Department of Chemical and Biological Sciences, Japan Women’s University, Tokyo; † Laboratory of Bioresources, National Institute for Basic Biology, The Graduate University for Advanced Studies (SOKENDAI), Aichi; ‡ National Institute for Basic Biology, Interuniversity Bio-Backup Project Center, Aichi, Japan

**Keywords:** medaka, oculocutaneous albinism type 2 (oca2), solute carrier family 45 member 2 (slc45a2), leucophore free, guanineless, iridophoreless-1

## Abstract

“See-through” strains of medaka are unique tools for experiments: their skin is transparent, and their internal organs can be externally monitored throughout life. However, see-through fish are less vital than normally pigmented wild-type fish, which allows only skilled researchers to make the most of their advantages. Expecting that hybrid vigor (heterosis) would increase the vitality, we outcrossed two see-through strains (SK^2^ and STIII) with a genetically distant wild-type strain (HNI). Fish with the see-through phenotypes were successfully restored in the F_2_ generation and maintained as closed colonies. We verified that genomes of these hybrid see-through strains actually consisted of approximately 50% HNI and approximately 50% SK^2^ or STIII alleles, but we could not obtain evidence supporting improved survival of larvae or fecundity of adults, at least under our breeding conditions. We also found that four of the five see-through mutations (*b^g8^*, *i-3*, *gu*, and *il-1* but not *lf*) additively decrease viability. Given that heterosis could not overwhelm the viability-reducing effects of the see-through mutations, easy-to-breed see-through strains will only be established by other methods such as conditional gene targeting or screening of new body-color mutations that do not reduce viability.

Body surfaces of vertebrates are pigmented by cells called chromatophores, up to six types of which (melanophore, xanthophore, erythrophore, leucophore, iridophore, and cyanophore) have been identified in fish (Fujii 2000). These intact chromatophores provide a powerful platform by which to study cell proliferation, differentiation, or migration (Hirobe 2011). However, when research focuses on other cells inside the body, these pigmented cells on the body surface become obstacles. This is particularly critical in experiments in which model fish are used (medaka and zebrafish) because their transparent somatic cells enable *in situ* observations of internal organs without dissection.

The “see-through” medaka no. 3 (STIII) was established from this point of view (Wakamatsu *et al.* 2001). It does not have any visible (fully differentiated) chromatophore throughout life, and therefore drug effects or fluorescent proteins expressed in the internal organs are maximally visualized (Kashiwada 2006; Deguchi *et al.* 2012). STIII is a quadruple recessive mutant with the following spontaneous body-color mutations: *albino-3* (*i-3*), *leucophore free* (*lf*), *guanineless* (*gu*), and *iridophoreless-1* (*il-1*; see Table 1). The *i-3* locus encodes the oculocutaneous albinism type 2 (Oca2) protein that is essential for melanin synthesis in melanophores (Fukamachi *et al.* 2004). Xanthophores in the *i-3* mutant are also colorless because of the potential function of Oca2 in carotenoid metabolism. The *lf* gene is essential for leucophore development, and the *lf* mutants lack any visible leucophores (Fukamachi *et al.* 2006). Proteins encoded by the *gu* or *il-1* locus are necessary for iridophore development in the eyes/abdomen or the opercles, respectively. STIII fish develop and grow normally (Iwamatsu *et al.* 2003) but are rather weak and difficult to breed, which has prevented widespread use of this strain in laboratories.

**Table 1:** An overview of the see-through mutations

Mutation*^a^*	Mutated Genes	Chromosomal Locations	Chromatophores Removed	Timing of the Phenotype Appearance	Introduced in
*i-3*	*Oculocutaneous albinism type 2*	LG04	Melanophore and xanthophore	From embryo to adult	STIII
*b^g8^*	*Solute carrier family 45*, *member 2*	LG12	Melanophore	From embryo to adult	SK^2^
*lf*	Unidentified	LG01	Leucophore	From embryo to adult	STIII and SK^2^
*gu*	Unidentified	LG05	Iridophore (in the eyes/abdomen)	From embryo to adult	STIII and SK^2^
*il-1*	Unidentified	Unidentified	Iridophore (in the opercles)	Adult only	STIII

a All mutations are recessive to the corresponding wild-type alleles.

We previously established another see-through strain, suke-suke (SK^2^), which is a triple recessive mutant of radiation-induced (*b^g8^*) and spontaneous (*lf* and *gu*) mutations (Fukamachi *et al.* 2008). The *b* locus, on which the *b^g8^* mutation locates, encodes the solute carrier family 45, member 2 (Slc45a2) protein that is essential for melanin synthesis (Fukamachi *et al.* 2001). The *b^g8^* mutation does not suppress melanin deposition as strongly as the *i-3* mutation (Fukamachi *et al.* 2004), and therefore the eyes (and xanthophores) of SK^2^ are weakly pigmented, unlike those of STIII.

The body-color mutations introduced into STIII or SK^2^ were isolated from a southern Japanese population [see (Fukamachi 2011)]. There is another (northern Japanese) population of medaka, which is so distantly related to the southern population that it was recently proposed as a different species [*Oryzias sakaizumii* (Asai *et al.* 2011)]. Genome sequences between the northern and southern populations are approximately 3% different, but their hybrids (F_1_s) are fully viable and fertile. Indeed, it is well known among medaka investigators that the F_1_ fish are full of vitality and extremely easy to handle. Therefore, we anticipated that outcrossing of the see-through southern strains with the northern strain and restoring F_2_ fish with the see-through phenotypes would result in hybrid vigor (heterosis) and establish easy-to-breed see-through strains that could tolerate widespread use in laboratories.

## Materials and Methods

### Fish and breeding conditions

We used STIII and SK^2^ as the southern see-through strains. As the northern wild-type strain, we used the standard inbred strain, HNI. All fish developed and were grown in the laboratory. Ordinary tap water heated at 27° was used and was circulated with a central filtration system. Light was provided from ordinary fluorescent lamps for 14 hr per day. We fed <2-week larvae with a few kinds of well-ground flake food (*e.g.*, TetraMin; Tetra), and older fish with live brine shrimps and the flake food, about five times per day (every 2 hr between 1000 and 1800 hr).

### Outcrossing and restoring F_2_ fish with the see-through phenotype

**Figure 1:**
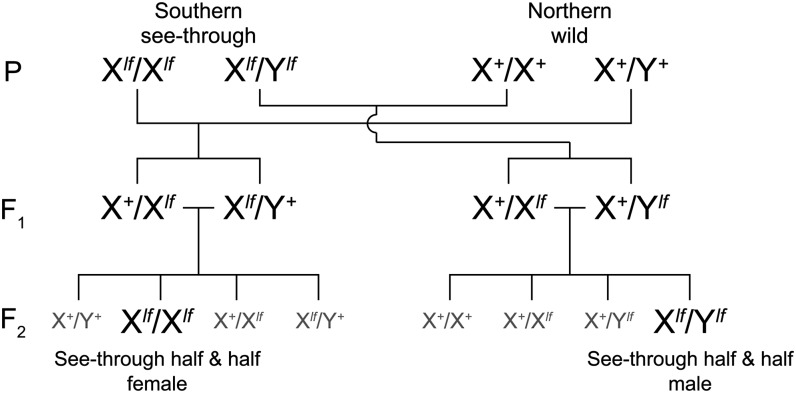
A schematic view of the outcrosses. Because the *lf* gene exists on both the X and Y chromosomes (LG01), reciprocal crosses between the see-through and wild-type parents (P) were necessary to obtain both sexes of see-through F_2_ fish whose genome consists of 50% northern and 50% southern alleles. The see-through mutations other than *lf* (*b^g8^*, *i-3*, *gu*, and *il-1*) are located on autosomes (see Table 1) and are omitted from this figure.

The *lf* gene is sex-linked and located on both the X and Y chromosomes (Wada *et al.* 1998). Therefore, we needed to cross the see-through and the wild-type fish reciprocally to obtain males and females with the see-through phenotype at the F_2_ generation (Figure 1).

As summarized in Table 1, all of the five see-through mutations (*b^g8^*, *i-3*, *lf*, *gu*, and *il-1*) are recessive, and all of the mutant phenotypes, except for *il-1*, appear from embryonic stages. Therefore, we could identify F_2_ embryos with the SK^2^ (*b^g8^-lf-gu* triple recessive) phenotype by binocular-microscopic observation and selectively breed these see-through F_2_s. In terms of STIII, we first selected F_2_ embryos with the *i-3-lf-gu* triple recessive phenotype (one-quarter of which should be the *i-3-lf-gu-il-1* quadruple recessive embryos) and raised all of the F_2_ progeny. When the *il-1* phenotype became apparent (about 2 mo after hatching), the triple and quadruple mutants were distinguished by intact or binocular-microscopic observations.

### Genome-wide genotyping

We randomly chose several adult fish from the original and hybrid see-through strains, and extracted their genomic DNA using a high-salt DNA extraction method (Aljanabi and Martinez 1997). Using each of the genomic DNA as templates, we amplified the M-marker 2009 using polymerase chain reaction and analyzed the bands as described elsewhere (Kimura and Naruse 2010). Because the sizes of all HNI alleles were already known (Kimura and Naruse 2010), we regarded bands at different sizes as the southern alleles.

### Vitality comparison

We focused on two characters to compare the vitality of the original and hybrid see-through strains: the survival rate of hatched larvae (viability) and the number of eggs daily spawned by adults (fecundity).

To assess viability, we collected fertilized eggs and placed hatched larvae into tanks (different strains in different tanks). When the number of larvae in the tanks reached 30, 43, or 50 (we could not obtain these numbers of hatched larvae in one day and needed to accumulate larvae hatched on different days but within the same week), we started counting live fish every week.

To assess fecundity, we prepared adult fish that were spawning every day, and we collected eggs attached to the females’ cloaca (spawned eggs are temporarily held at the cloaca by attaching filaments) and those that dropped on the bottom of the tanks every morning. Fertilized and unfertilized eggs were distinguished and counted under a binocular microscope. We incubated the fertilized eggs in methylene-blue-added tap water until they hatched.

### Assessment of the see-through mutations on viability

We backcrossed the F_1_ females (Figure 1) with the original see-through males to obtain embryos with various body-color phenotypes (*i.e.*, wild-type and single/double/triple/quadruple recessive mutants) in the same numbers; the *b*, *i-3*, *lf*, and *gu* loci are independent on chromosomes (Naruse *et al.* 2000; Fukamachi *et al.* 2004), and the *il-1* locus seemed not to be linked to any of these loci (see Wakamatsu *et al.* 2001 and the *Results* section). It should be noted that this cross (but not the reciprocal cross) produces siblings of both sexes in all of the phenotypes. Each embryo was phenotyped for the *b*, *i-3*, *lf*, and *gu* loci, and hatched larvae were raised *en masse* in large containers [51 cm × 36 cm × 24 cm (length, width, height)] without water circulation/filtration. When they reached adult stages, we rephenotyped each fish for all of the five see-through loci, including *il-1*. The body length (from the snout to the distal edge of the caudal fin) of all the adult fish was also measured.

## Results and Discussion

### Outcross of SK^2^ and STIII

We hybridized the southern see-through strains (SK^2^ and STIII) with a northern wild-type strain (HNI). HNI is one of the standard inbred strains widely used in laboratories and has wild-type alleles at all of the five see-through loci (*b*, *i-3*, *lf*, *gu*, and *il-1*). Their F_1_s, which were heterozygous for all of these loci, exhibited the wild-type phenotype. We intercrossed the F_1_ fish and collected a large number of F_2_ siblings, among which we screened individuals with the SK^2^ or STIII phenotype (Figure 1).

**Figure 2:**
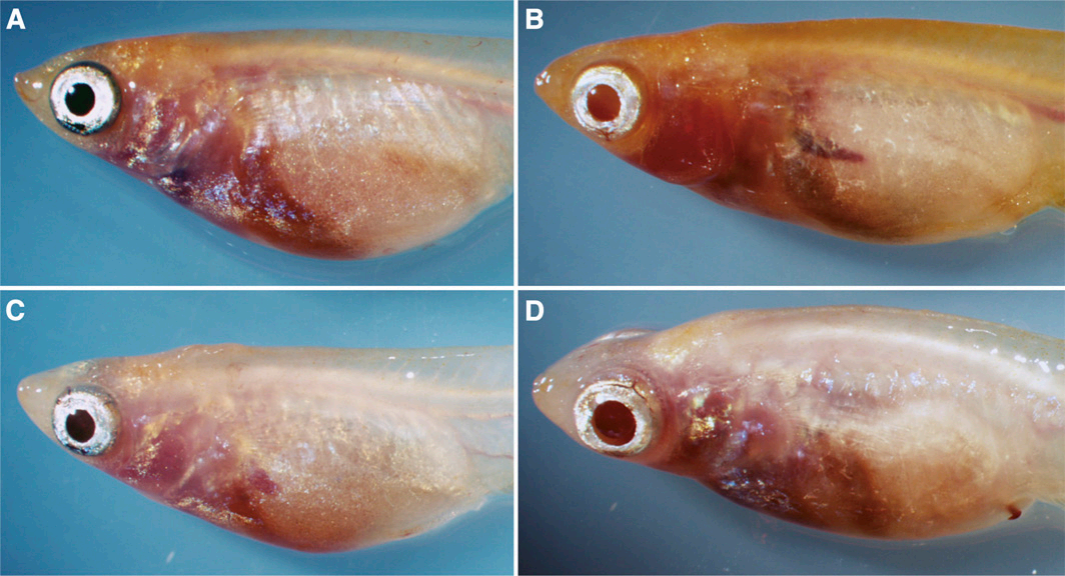
The see-through strains analyzed in this study. (A) SK^2^, (B) STIII, (C) SK^2^-HH, and (D) STII-HH. Note the increased transparency of the opercles and the anterior abdomen of STIII and the slightly melanized eyes of SK^2^ and SK^2^-HH in comparison with those of STIII and STII-HH.

All of the SK^2^ mutations (*b^g8^*, *lf*, and *gu*) are independently located on chromosomes (Naruse *et al.* 2000), and therefore 1/4^3^ (= 1/64) of the F_2_ fish should develop the SK^2^ phenotype. Among the 4969 F_2_ eggs that we collected, 4880 (98.2%) developed normally (note this extremely high fertility/hatch rate demonstrating the full vitality of the hybrids [F_1_ adults and F_2_ embryos]). Sixty-seven of the embryos exhibited the SK^2^ phenotype, which was not significantly different from the expected value (4880 × 1/64 = 76.25; *P* = 0.286, χ^2^ test). Discarding all other embryos, we selectively raised these see-through F_2_s and could obtain 24 male and 13 female adult fish. F_3_ and later generations were successfully maintained as a closed colony by interbreeding. Because the genome of this newly established see-through strain should theoretically consist of approximately 50% northern and approximately 50% southern alleles, we termed the strain SK^2^ half-and-half (SK^2^-HH; Figure 2).

The *i-3*, *lf*, and *gu* loci of the STIII mutations are independent (Naruse *et al.* 2000; Fukamachi *et al.* 2004), but the location of the *il-1* locus is unknown. If the *il-1* mutation is not linked to any of the other STIII mutations, F_2_ fish with the STIII phenotype should appear at a frequency of 1/4^4^ (= 1/256). By contrast, if the *il-1* mutation is (closely) linked to either of the other STIII mutations, such F_2_ should be obtained at a higher frequency. Among the 8,011 F_2_ eggs that we collected, 7,723 (96.4%) embryos developed normally, 94 of which exhibited the *i-3-lf-gu* triple recessive phenotype (the *il-1* phenotype does not appear at this stage). Interestingly, this count was close to, but significantly lower than, the expected value (7,723 × 1/64 = 120.7; *P* = 0.014, χ^2^ test). The reason is unclear, but one or a combination of the STIII mutations may slightly inhibit normal development of embryos, which could statistically be detected only when thousands are examined (note that such an effect was not detected in Figure 5A, where only 773 embryos were examined). We selectively raised these triple recessive F_2_ larvae, obtained 34 adult fish, and found only two males and two females with the STIII (*i-3-lf-gu-il-1* quadruple recessive) phenotype. This count (*i.e.*, four) was also significantly lower than the expected value (34 × 1/4 = 8.5; *P* = 0.017, χ^2^ test), which most likely reflects the viability-reducing effect of the *il-1* mutation (described later; see Figure 5). This low count of the quadruple recessive F_2_s also indicates that the *il-1* locus is not closely linked to the *i-3*, *lf*, or *gu* loci.

Unfortunately, we could not obtain fertilized eggs from the quadruple recessive F_2_ males and females, and we could not establish the STIII-HH strain. Therefore, we intercrossed their triple recessive (*i-3-lf-gu*) siblings and maintained the strain as a closed colony. This strain seemed to retain the *il-1* mutation in the population, but the majority (*e.g.*, 12/13) exhibited a triple recessive phenotype known as see-through medaka no. 2 [STII; (Wakamatsu *et al.* 2001)], and we termed the strain STII-HH (Figure 2).

### Genome-wide genotyping of the see-through-HH strains

We then investigated whether the genomes of SK^2^-HH and STII-HH actually consist of half northern and half southern alleles. For this purpose, we used the M-marker 2009, which is a set of 48 sequence-tagged sites (STSs) that were designed at all of the 24 medaka chromosomes (two markers on each chromosome). Polymorphisms in these STSs can be detected as insertions or deletions by genomic polymerase chain reaction and capillary electrophoresis (Kimura and Naruse 2010).

When we analyzed the original SK^2^ and STIII strains (three males and three females for each strain; n = 6 each), we found that their genomes did not have a northern (HNI) allele at any loci (Table 2). By contrast, genomes of SK^2^-HH (six males and eight females; n = 14) and STII-HH (seven males and five females; n = 12) did contain the northern alleles (Table 2). Among the 96 alleles amplified by the M-marker 2009 in each fish, 49.8 ± 2.3% and 41.6 ± 1.4% (mean ± SEM) were northern in SK^2^-HH and STII-HH, respectively. Thus, the outcrosses successfully introduced the northern alleles into the see-through-HH strains and their genomes literally consisted of half northern and half southern alleles.

**Table 2:** Genome scanning using the M-marker 2009

Linkage Group	STS	Genotype Frequency (N/N homo: N/S hetero: S/S homo [ND])			Genes Located
		SK^2^/STIII (n = 6 each)	SK^2^-HH (n = 14)	STII-HH (n = 12)	
LG01	MID0121	0: 0: 12 [0]	2: 7: 5 [0]	0: 4: 8 [0]*^a^*	*lf*
	MID0117	0: 0: 12 [0]	0: 0: 14 [0]*^a^*	0: 0: 12 [0]*^a^*	
LG02	MID0225	0: 0: 12 [0]	3: 11: 0 [0]	6: 6: 0 [0]	
	MID0222	0: 0: 12 [0]	9: 4: 1 [0]*^a^*	3: 8: 1 [0]	
LG03	MID0313	0: 0: 12 [0]	4: 6: 4 [0]	1: 11: 0 [0]	
	MID0316	0: 0: 12 [0]	7: 4: 3 [0]	3: 9: 0 [0]	
LG04	MID0414	0: 0: 12 [0]	6: 8: 0 [0]	0: 6: 6 [0]	*i-3*
	MID0424	0: 0: 12 [0]	2: 12: 0 [0]	0: 0: 12 [0]*^a^*	
LG05	MID0514	0: 0: 12 [0]	1: 11: 2 [0]	0: 1: 11 [0]*^a^*	*gu*
	MID0517	0: 0: 12 [0]	0: 5: 9 [0]*^a^*	0: 0: 12 [0]*^a^*	
LG06	MID0602	0: 0: 12 [0]	5: 9: 0 [0]	0: 9: 3 [0]	
	MID0621	0: 0: 12 [0]	3: 10: 1 [0]	0: 8: 4 [0]	
LG07	MID0703	0: 0: 12 [0]	5: 9: 0 [0]	0: 5: 7 [0]	
	MID0706	0: 0: 12 [0]	5: 6: 3 [0]	1: 7: 4 [0]	
LG08	MID0812	0: 0: 12 [0]	0: 2: 7 [5]*^a^*	0: 5: 7 [0]	
	MID0822	0: 0: 12 [0]	2: 7: 4 [1]	0: 6: 6 [0]	
LG09	MID0913	0: 0: 12 [0]	5: 6: 3 [0]	0: 6: 6 [0]	
	MID0916	0: 0: 12 [0]	1: 10: 3 [0]	4: 4: 4 [0]	
LG10	MID1012	0: 0: 12 [0]	2: 11: 2 [0]	5: 4: 3 [0]	
	MID1014	0: 0: 11 [1]	5: 8: 1 [0]	5: 4: 3 [0]	
LG11	MID1112	0: 0: 12 [0]	5: 9: 0 [0]	5: 7: 0 [0]	
	MID1116	0: 0: 12 [0]	1: 9: 4 [0]	6: 3: 3 [0]	
LG12	MID1213	0: 0: 12 [0]	0: 6: 8 [0]*^a^*	3: 5: 4 [0]	*b*
	MID1221	0: 0: 12 [0]	0: 6: 8 [0]*^a^*	4: 5: 3 [0]	
LG13	MID1303	0: 0: 12 [0]	0: 10: 4 [0]	0: 7: 5 [0]	
	MID1306	0: 0: 12 [0]	3: 10: 1 [0]	0: 8: 4 [0]	
LG14	MID1422	0: 0: 12 [0]	1: 9: 4 [0]	5: 7: 0 [0]	
	MID1414	0: 0: 12 [0]	5: 8: 1 [0]	5: 7: 0 [0]	
LG15	MID1512	0: 0: 12 [0]	3: 10: 1 [0]	1: 5: 6 [0]	
	MID1505	0: 0: 12 [0]	5: 9: 0 [0]	0: 7: 5 [0]	
LG16	MID1602	0: 0: 12 [0]	6: 8: 0 [0]	2: 5: 5 [0]	(*il-1*?)
	MID1614	0: 0: 12 [0]	3: 8: 3 [0]	0: 1: 11 [0]*^a^*	
LG17	MID1713	0: 0: 11 [1]	1: 8: 5 [0]	0: 11: 1 [0]	
	MID1718	0: 0: 11 [1]	4: 6: 4 [0]	7: 4: 1 [0]	
LG18	MID1826	0: 0: 11 [1]	7: 7: 0 [0]	1: 9: 2 [0]	
	MID1807	0: 0: 12 [0]	9: 5: 0 [0]*^a^*	9: 3: 0 [0]*^a^*	
LG19	MID1924	0: 0: 12 [0]	4: 7: 3 [0]	0: 7: 5 [0]	
	MID1915	0: 0: 12 [0]	3: 7: 4 [0]	0: 7: 5 [0]	
LG20	MID2003	0: 0: 11 [1]	3: 8: 2 [1]	4: 8: 0 [0]	
	MID2015	0: 0: 12 [0]	2: 10: 2 [0]	0: 10: 2 [0]	
LG21	MID2113	0: 0: 12 [0]	6: 0: 8 [0]*^a^*	7: 0: 5 [0]*^a^*	
	MID2105	0: 0: 12 [0]	1: 9: 4 [0]	0: 4: 8 [0]*^a^*	
LG22	MID2213	0: 0: 12 [0]	1: 10: 3 [0]	4: 6: 2 [0]	
	MID2215	0: 0: 12 [0]	0: 9: 5 [0]	2: 5: 5 [0]	
LG23	MID2311	0: 0: 12 [0]	3: 8: 2 [1]	1: 9: 2 [0]	
	MID2314	0: 0: 12 [0]	1: 9: 3 [1]	3: 8: 1 [0]	
LG24	MID2412	0: 0: 12 [0]	1: 9: 3 [1]	3: 8: 1 [0]	
	MID2425	0: 0: 12 [0]	0: 8: 6 [0]	0: 10: 2 [0]	

STS, sequence-tagged sites; N, northern allele; S, southern allele; ND, not determined.

a Significant difference from the expected value of 1:2:1 in SK^2^-HH and STII-HH (*P* < 0.01, χ^2^ test without correction).

Given the allele frequencies in the see-through-HH strains noted above, the genotype frequency in each marker could be expected to be northern-homozygous (N/N):heterozygous (N/S):southern-homozygous (S/S) = 1:2:1 on the assumption of the Hardy–Weinberg equilibrium. However, there are several DNA markers in Table 2 that do not fit with this expectation (*P* < 0.01, χ^2^ test without correction). Nevertheless, we could consider plausible reasons for most of these biased genotype frequencies.

For example, only the S/S genotype was detected in MID0117 on LG01 in both SK^2-^HH and STII-HH. This is most likely because the *lf* locus is located on LG01 (see Table 1). That is, the mutated *lf* allele is of southern origin, and the *lf/lf* genotype was selected to establish SK^2^-HH and STII-HH, which should fix the southern alleles of STSs/genes flanking to the *lf* locus (including MID0117) in these strains. The same should be the case in MID0517 on LG05, where the *gu* locus is located.

The biased genotype frequency was detected in either (not both) of SK^2^-HH or STII-HH in terms of MID0424 on LG04 and MID1213/1221 on LG12. This is because the *i-3* mutation on LG04 was fixed only in STII-HH (but not in SK^2^-HH) and the *b^g8^* mutation on LG12 was fixed only in SK^2^-HH (but not in STII-HH). From this point of view, the genotype frequency of MID1614 on LG16, which is strongly biased in only STII-HH, but not SK^2^-HH, may indicate that the *il-1* locus is located on this linkage group (though the mutation has not been completely fixed in the strain).

The genotype frequency of MID1807 on LG18 is differently biased from the cases described previously in that its northern (instead of southern) allele was more frequently detected in both SK^2^-HH and STII-HH. The reason remains unknown, but northern alleles of the chromosomal region around MID1807 may be more advantageous for survival and/or reproduction than southern alleles, which therefore were selected in both strains.

Another interesting genotype frequency is found in MID2113 on LG21, where the heterozygous N/S never appeared in either SK^2^-HH or STII-HH. Considering that we used adult fish (instead of embryos) for genotyping, N/S heterozygotes of this chromosomal region may not be able to survive or grow efficiently because of incompatibility between northern and southern alleles (outbreeding depression).

### Vitality of the see-through-HH strains

**Figure 3:**
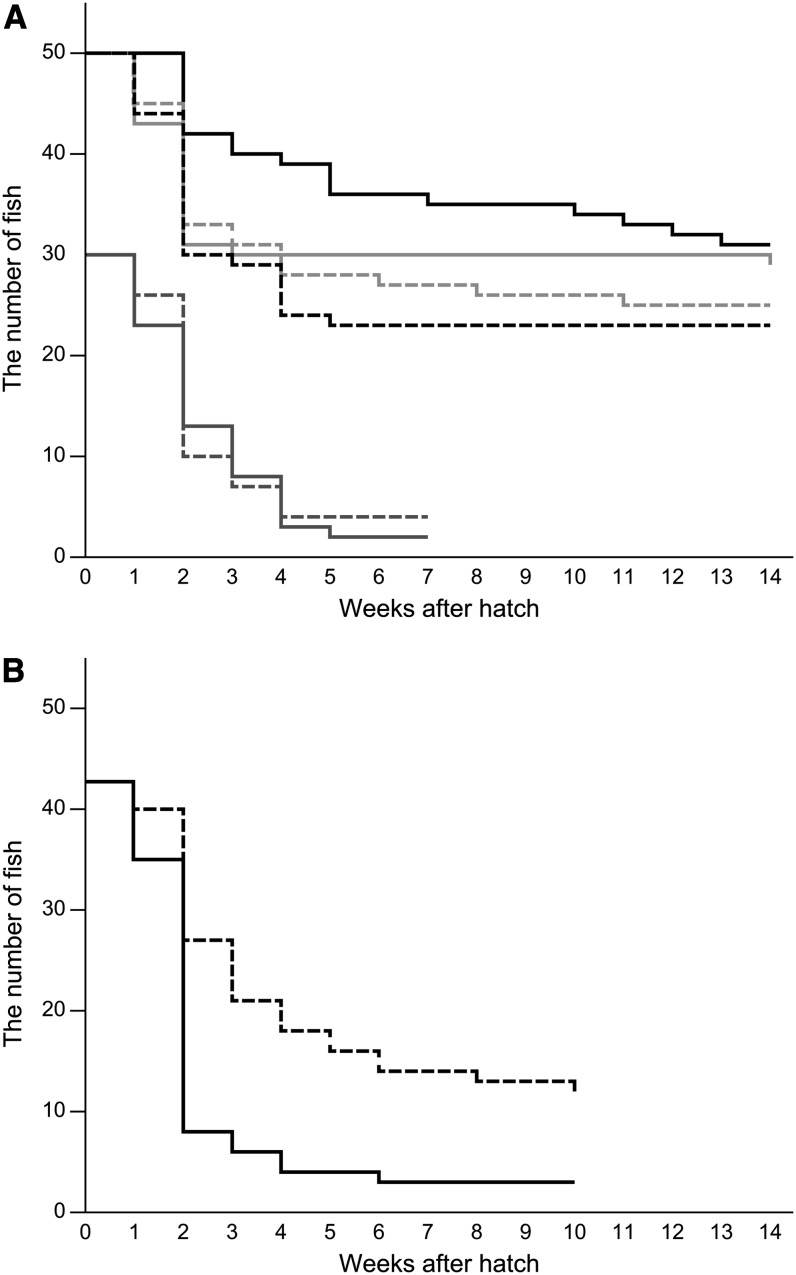
Survival curves of the see-through strains. (A) Comparison between SK^2^ and SK^2^-HH. Three independent experiments were performed, which were distinguished by colors (light gray and black starting at 50, and light gray starting at 30). Lines and dotted lines represent SK^2^ and SK^2^-HH, respectively. (B) Comparison between STIII and STII-HH. Lines and dotted lines represent STIII and STII-HH, respectively.

The most critical period when breeding medaka is up to 2 wk after hatching; after surviving this period, most fish can grow into adults (see Figure 3). We could stably maintain both SK^2^ and SK^2^-HH in the laboratory (see the section *Materials and Methods* for our breeding conditions), but we did not have the impression that SK^2^-HH was particularly easier to handle than SK^2^; many larvae of both strains died after hatching. Indeed, survival rates (up to 14 wk after hatch) were not significantly different between SK^2^ and SK^2^-HH in any of the three independent comparisons (Figure 3A; *P* > 0.05, logrank test with Bonferroni correction). We also measured the body length of all the survivors in the first and second comparisons (in which the observation was continued for 14 weeks) and detected no significant difference between the strains (24.5 ± 0.3 mm [n = 60] in SK^2^ and 25.4 ± 0.5 mm [n = 48] in SK^2^-HH; *P* = 0.108, Student’s two-tailed *t*-test).

The fact that we twice failed to maintain STIII, but not STII-HH, should indicate that STII-HH is easier to breed than STIII. Indeed, the survival rate of STII-HH was four times greater than that of STIII, at least in one comparison (Figure 3B; *P* < 0.001, log-rank test). However, this improvement seemed to reflect not heterosis but removal of the viability-reducing *il-1* mutation in STII-HH (explained below), and we did not repeat the comparison.

We also compared the fecundity of SK^2^ and SK^2^-HH (Table 3). We prepared four pairs of adult fish from each strain that were spawning every morning and collected their eggs for 7 consecutive days. The body lengths of the females were 33.2 ± 0.8 mm and 33.7 ± 1.0 mm in SK^2^ and SK^2^-HH, respectively (n = 4 each), which were not significantly different (*P* = 0.691, Student’s two-tailed *t*-test). The total numbers of the collected eggs were 300 for SK^2^ and 324 for SK^2^-HH, which is a ratio not significantly different from 1:1 (*P* = 0.337, χ^2^ test). The numbers of fertilized eggs were 155 and 135, and the numbers of hatched larvae were 131 and 107 for SK^2^ and SK^2^-HH, respectively, neither of which is a ratio significantly different from 1:1 (*P* > 0.05, χ^2^ test). In short, only about five fertilized eggs (and four hatched fries) could be obtained per female per day in both SK^2^ and SK^2^-HH of this body size.

**Table 3:** Fecundity of SK^2^ and SK^2^-HH

Strain	Body Length of Females (n = 4), mm	Total no. of Eggs Collected in 7 Contiguous Days^1^	No. Fertilized Eggs^2^	Fertility Rate, %*^a^*	No. Hatched Larvae^3^	Hatching Rate, %*^b^*
SK^2^	33.2 ± 0.8	300	155	51.7%	131	84.5
SK^2^-HH	33.7 ± 1.0	324	135	41.7%	107	79.3

a ^2/1^.

b ^3/2^.

Taken together, we could not obtain evidence supporting the notion that the hybrid see-through strains were any easier to handle than the original see-through strains (Figure 3 and Table 3) despite the fact that the genomes of the see-through-HH strains actually consisted of a 1:1 mixture of the northern and southern alleles (Table 2). These results would indicate that inbreeding depression (Charlesworth and Willis 2009) seldom occurred in the original SK^2^ or STIII strains. That is, although all of the alleles that we detected in SK^2^ and STIII were southern in size (Table 2), their genomes must be sufficiently heterozygotic to avoid the depression. Alternatively, effects of the heterosis might actually exist in the see-through-HH strains but might be masked and overlooked in the present study. Our breeding conditions, in which STIII could not be stably maintained, are not maximally optimized, and we did not test other breeding conditions (*e.g.*, food, fish densities, or water flow; see Hensley and Leung 2010). Therefore, the masked heterosis may be manifested if similar experiments are performed under different breeding conditions.

### Effects of the see-through mutations on viability

Given that the see-through-HH strains did not show improved viability or fecundity (at least under our breeding conditions), the decreased vitality of the original and hybrid see-through strains should most likely be the result of the see-through mutations themselves. To assess this hypothesis, we backcrossed the F_1_ fish (Figure 1) to the original see-through fish, raised the backcrossed siblings to adult stages *en masse*, and examined the phenotype and body length of all survivors.

**Figure 4:**
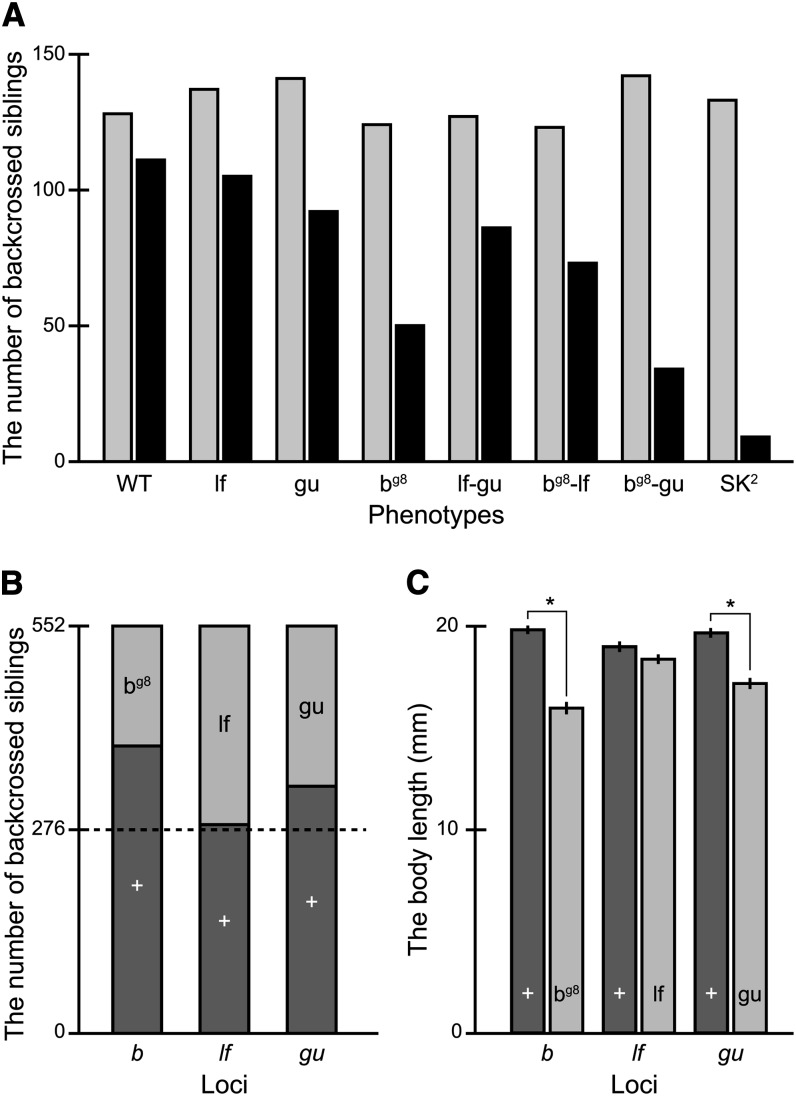
Assessment of the SK^2^ mutations on viability. (A) The numbers of backcrossed siblings (obtained by crossing F_1_ females to SK^2^ males) with various phenotypes at embryonic (gray) and adult (black) stages. (B) All of the 552 adult siblings obtained in panel A were divided into two groups according to either the *b^g8^*, *lf*, or *gu* phenotypes, and the numbers of individuals with wild-type and mutant phenotypes are shown as dark- and light-gray bars, respectively. (C) Body length of the wild-type (dark gray) and mutant (light gray) groups classified in panel B. Asterisks indicate significant differences (*P* < 0.001, Student’s two-tailed *t*-test). Note the decreased number (B) and body length (C) in the *b^g8^* and *gu* but not *lf* mutant siblings.

From the SK^2^ backcross, we obtained a total of 1047 hatched larvae, whose embryonic phenotypes had been determined under a binocular microscope at day 5–6 after fertilization. As expected from the independent location of the *b*, *lf*, and *gu* genes (Naruse *et al.* 2000), the backcrossed larvae exhibited eight kinds of body-color phenotypes (wild-type, *b^g8^*, *lf*, *gu*, *b^g8^-lf*, *b^g8^-gu*, *lf-gu*, and *b^g8^-lf-gu*) in equal numbers (1047 × 1/2^3^ ≅ 131; Figure 4A; *P* = 0.885, χ^2^ test test). About 2 mo after their mixed breeding using three large containers (see *Materials and Methods*), a total of 552 fish had survived (a survival rate of 52.7%), but the numbers were no longer equal among the phenotypes (*P* < 0.001, χ^2^ test; Figure 4A); fish with more mutations tended not to survive as well as those with fewer mutations. That is, only eight fish with the triple-recessive phenotype survived, whereas 110 of their wild-type siblings survived in the identical containers.

To analyze the data further, we classified these 552 survivors into two groups according to presence or absence of either of the *b^g8^*, *lf*, and *gu* phenotypes, and we compared the numbers (Figure 4B). The number of wild-type siblings was significantly higher than that of mutant siblings (*P* < 0.001, χ^2^ test), when the survivors were grouped according to the *b^g8^* or *gu* phenotype. By contrast, when the survivors were grouped according to the *lf* phenotype, the numbers of wild-type and mutant siblings were not significantly different (*P* = 0.551, χ^2^ test). These results demonstrate that the *b^g8^* and *gu* mutations, but not the *lf* mutation, significantly decrease the probability of larvae growing into adults. Furthermore, considering that siblings with both of the *b^g8^* and *gu* mutations (*i.e.*, *b^g8^-gu* and *b^g8^-lf-gu*) survived less than those with either of the mutations (*i.e.*, *b^g8^*, *gu*, *b^g8^-lf*, and *lf-gu* siblings; Figure 4A), the viability-reducing effect of the *b^g8^* and *gu* mutations seems to function additively.

Our data also showed that the *b^g8^* and *gu* mutations suppress growth; siblings without these mutations grew significantly larger than those with the mutations (*P* < 0.001, Student’s two-tailed *t*-test; Figure 4C). Again, such an effect was not detected in the *lf* mutation (*P* = 0.097; Figure 4C).

**Figure 5:**
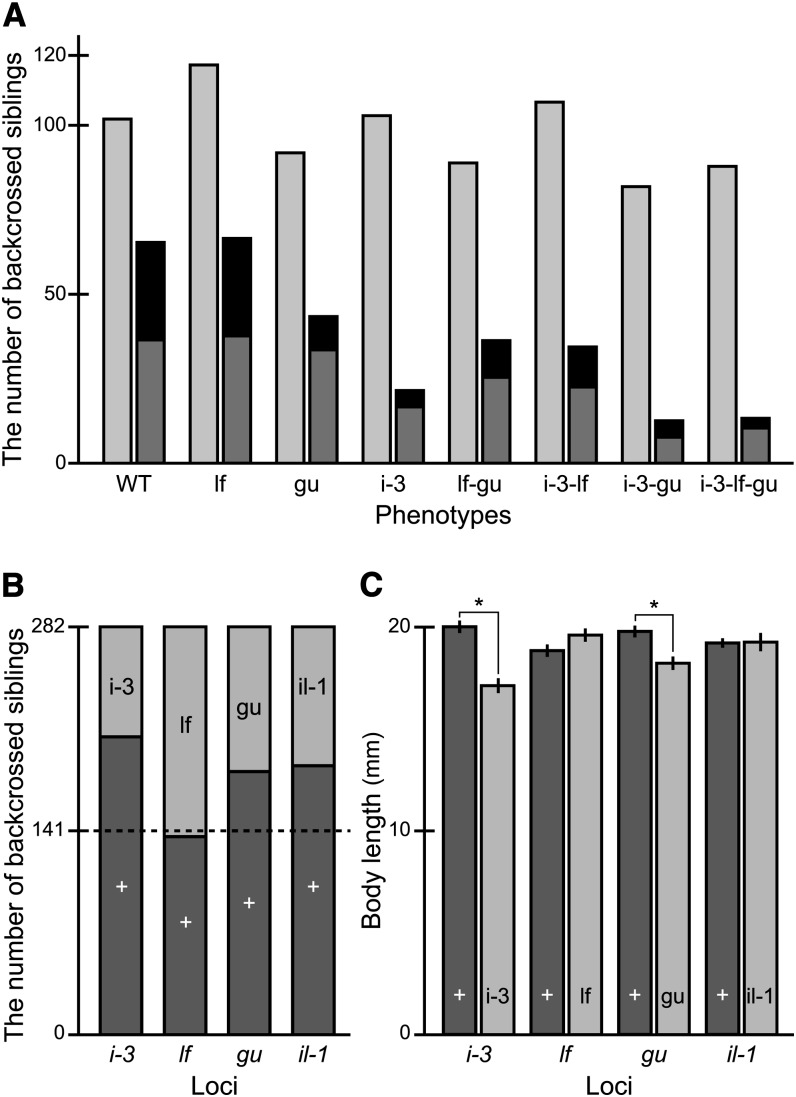
Assessment of the STIII mutations on viability. (A) The numbers of backcrossed siblings (obtained by crossing F_1_ females to STIII males) with various phenotypes at embryonic (light gray) and adult (dark gray and black) stages. Because the *il-1* phenotype only appears in adult stages, embryos were classified into eight groups. Adults were classified into 16 groups as shown by dark gray (not accompanied by the *il-1* phenotype) and black (accompanied by the *il-1* phenotype). (B) All of the 282 adult siblings obtained in panel A were divided into two groups according to either the *i-3*, *lf*, *gu*, or *il-1* phenotypes, and the numbers of individuals with wild-type and mutant phenotypes are shown as dark- and light-gray bars, respectively. (C) Body length of the wild-type (dark-gray) and mutant (light-gray) groups classified in panel B. Asterisks indicate significant differences (*P* < 0.001; Student’s two-tailed *t*-test). Note the decreased number in *i-3*, *gu*, and *il-1* (but not *lf*) siblings (B), and the decreased body length in *i-3* and *gu* (but not *lf* or *il-1*) siblings (C).

From the STIII backcross, we obtained a total of 773 hatched larvae, which had been phenotyped for the *i-3*, *lf*, and *gu* (but not *il-1*) loci during embryonic stages. Supporting their independent locations on chromosomes (Table 1), eight phenotypes appeared in equal numbers (773 × 1/2^3^ ≅ 97; *P* = 0.173, χ^2^ test; Figure 5A) in the backcrossed siblings. After two months of their breeding *en masse*, we obtained 282 adult fish (a survival rate of 36.5%), phenotyped each of them for the *i-3*, *lf*, *gu*, and *il-1* loci and classified them into 16 groups (Figure 5A). As for the case of the SK^2^ backcross, the numbers of survivors apparently differ among the groups (*P* < 0.001, χ^2^ test); *e.g.*, we obtained only two quadruple-recessive fish, whereas 36 wild-type siblings survived in the same environment.

Classification of the 282 survivors into wild-type and mutant groups according to either of the *i-3*, *lf*, *gu*, and *il-1* loci revealed that siblings with the *i-3*, *gu*, or *il-1* phenotypes, but not the *lf* phenotype, survived less than their corresponding wild-type siblings. It is noteworthy that the results for *lf* and *gu* were consistent between the SK^2^ and STIII backcrosses (Figures 4B and 5B). The growth-suppressing effect of *gu*, but not *lf*, detected in the SK^2^ backcross (Figure 4C) also was reproduced in the STIII backcrosses (Figure 5C), and the *i-3* mutation was shown to have the same growth-suppressing effect (Figure 5C). It is intriguing that—unlike *b^g8^*, *gu*, and *i-3*—the *il-1* mutation, which reduced viability (Figure 5B), did not suppress growth (*P* = 0.936, Student’s *t*-test; Figure 5C). Therefore, the mechanism by which the *il-1* mutation reduces viability should be different from that of the *b^g8^*, *i-3*, and *gu* mutations (discussed below).

### Potential mechanisms by which the see-through mutations reduce viability

It was understandable that the *b^g8^* and *i-3* mutations reduced viability because these mutations suppress melanin deposition in the eyes (and skin), causing a typical phenotype known as albino. The *Slc45a2* and *Oca2* genes, on which the *b^g8^* and *i-3* mutations locate in medaka (Table 1), are also found in humans and are mutated in oculocutaneous albinism type 4 and 2 (OCA4 and OCA2) patients, respectively (Suzuki and Tomita 2008). Because OCA patients face several problems in visual acuity because of their amelanotic eyes (Gronskov *et al.* 2007), we suspect that the *b^g8^* or *i-3* fish might have similar optical problems. These albino fish would not be able to find and catch food efficiently in tanks, which would cause malnutrition, suppress growth (Figures 4C and 5C), and reduce viability (Figures 4B and 5B). However, our data do not exclude the possibility that the growth suppression and reduced viability are directly caused by pleiotropic effects of these albino mutations (such as diminishing food appetite, reducing nutrient absorption from guts, or preventing anabolism).

Considering that the *gu* mutants showed the same defects in growth and viability (Figures 4, B and C, and 5, B and C), we suspect that the *gu* mutation causes optical problems similar to those of *b^g8^* and *i-3*. The eyes of wild-type medaka (and many other fish species) are surrounded by a dense distribution of iridophores, which make them iridescent and silver in color when exposed to light. Because the *gu* mutation removes many of the iridophores, the amount of light coming into the eyes or light reflections inside the eyes would not be appropriately controlled. Considering that the *gu* mutation and the *b^g8^* and *i-3* mutations affect different types of chromatophores, the *b^g8^-gu* and *i-3-gu* double mutants should face more crucial optical problems than the *b^g8^*, *i-3*, or *gu* single mutants, and this appears to be detected as the additive effects of these mutations on the larval viability (Figure 4A and Figure 5A).

It was surprising to us that the *il-1* mutation, which only suppresses iridophore distribution on the opercles (Figure 2), apparently reduced viability (Figure 5B). This effect should not occur in the same mechanism as that of *b^g8^*, *i-3*, and *gu* (*i.e.*, optical problems leading to malnutrition), because the *il-1* fish grow as big as their wild-type siblings (Figure 5C). The definite mechanism remains unknown, but the iridophores on the opercles may have an indispensable role in protecting the gills (and/or surrounding tissues) from light exposure, and fish may die young without the protection against this phototoxicity. To our knowledge, however, the negative relationship between light exposure on the gills (or other internal organs) and organismal viability has not been elucidated to date. Alternatively, the *il-1* gene may have pleiotropic functions other than the iridophore development that are essential for retaining viability. It is also possible that not the *il-1* mutation but one or more other mutations closely linked to the *il-1* locus might be the actual cause of the reduced viability (genetic hitchhiking). Cloning and characterization of the *il-1* gene would open up a way to assess these possibilities.

### Future establishment of an easy-to-breed see-through strain

Since the establishment of SK^2^ (Fukamachi *et al.* 2008), we have had an impression that it is much easier to breed than STIII. We believe that this is because the *b^g8^* mutation suppresses melanin deposition in the eyes less severely than the *i-3* mutation (Figure 2; Fukamachi *et al.* 2004). Indeed, SK^2^, but not STIII larvae, could be raised outdoors (K. Naruse and S. Fukamachi, unpublished data), indicating that STIII with their less-pigmented eyes face more optical troubles than SK^2^ under strong light conditions. Under the present indoor conditions, however, the *b^g8^* and *i-3* mutations seemed to reduce viability to a similar degree, because the wild-type-to-mutant ratios of the backcrossed survivors (Figures 4B and 5B) were 71:29 and 73:27 when the fish were grouped based on the *b^g8^* and *i-3* phenotypes, respectively. Therefore, the aforementioned scenario would most likely reflect the fact that SK^2^ has only two viability-reducing mutations (*b^g8^* and *gu*), whereas STIII has three (*i-3*, *gu*, and *il-1*).

Given that the outcrosses did not increase the vitality of the see-through strains and that four of the five see-through mutations reduced viability (Figures 3–5, Table 3), an easy-to-breed see-through strain will only be established by adopting more complex methods. One such method could include screening of body-color mutations that do not decrease vitality. This study has already revealed that one of the four types of chromatophores in medaka, leucophores, can be perfectly removed by the *lf* mutation without reducing viability or suppressing growth (Figures 4, B and C, and 5, B and C). However, to eliminate the other three types (melanophores, iridophores, and xanthophores), screening of viable mutations other than *b^g8^*, *i-3*, *gu*, and *il-1* is necessary. Such mutations may already exist in mutant stock, such as the Tomita collection (Kelsh *et al.* 2004) or commercially available strains.

Genetic engineering is another possible method. Transgenesis and gene targeting are applicable in medaka (Ishikawa *et al.* 2010; Yan *et al.* 2013). Therefore, identification/utilization of *cis*-regulatory elements to express the *b*, *i-3*, and *gu* gene specifically in the eyes may solve optical problems and enhance growth and viability of the see-through strain.

The *il-1* phenotype was originally described as having an absence of iridophores in the opercles. Against the *gu* background, however, it becomes apparent that the *il-1* mutation also removes iridophores in the anterior part of the abdomen (Figure 2), and therefore it should be better when introduced into see-through strains to increase transparency. However, the mutation (or a hitchhiking mutation/s) reduces viability by an unpredictable mechanism (Figure 5B). If it is a hitchhiking mutation that reduces viability or if the *il-1* gene has other pleiotropic functions that directly affect viability, genetic manipulation would enable removal of the iridophores without reducing viability. However, if the phototoxicity in the gill or surrounding tissues/organs is the cause of the reduced viability, a reduction in viability (Figure 5B) seems to be inevitable. Breeding under dim light (or in darkness) may be a solution, but such a strain will not be easy to breed in laboratories with ordinary breeding apparatuses. Establishment of optimized and simplified protocols would be another effective approach for breeding see-through strains (Hensley and Leung 2010).

In summary, considering that the genomic background (and consequently the phenotype) of the see-through-HH strains is less uniform than that of the original strains (Table 2), we propose that the original SK^2^ is the most recommended easy-to-breed see-through strain at this time. This fish is now available at the National Bioresource Project (NBRP) Medaka upon request, and we hope that this availability expands opportunities for investigators.
